# Competitive tenders on analogue hospital pharmaceuticals in Denmark 2017–2020

**DOI:** 10.1186/s40545-022-00464-6

**Published:** 2022-10-22

**Authors:** Lars Holger Ehlers, Morten B. Jensen, Henrik Schack

**Affiliations:** 1 Nordic Institute of Health Economics, Aarhus, Denmark; 2 Danish Center for Healthcare Improvements, Faculty of Social Sciences and Faculty of Health Sciences, Aalborg University, Aalborg, Denmark; 3 Department of Economics and Business Economics, Aarhus University, Aarhus, Denmark; 4 Department of Health Science and Technology, Aalborg University, Aalborg, Denmark

**Keywords:** Tender system, Analogue competition healthcare cost, Cost-containment, Competitive tenders on pharmaceuticals, Public procurement, Managing access to medicine

## Abstract

**Background:**

Competitive tenders on pharmaceuticals are one of the most effective cost-containment instruments in healthcare systems. Its effectiveness has been demonstrated, among other things, in markets for generic medicine and biosimilars. In Denmark, an internationally unique model for competitive tenders on analogue substitutable pharmaceuticals has been developed and implemented for all public hospitals.

**Methods:**

We obtained data on all analogue competitive tenders carried out by the Danish Medicines Council from its foundation on January 1, 2017, to October 9, 2020. We calculated univariate descriptive statistics, pairwise correlations and made a multiple regression analysis on tender savings.

**Results:**

Average annual saving on hospital pharmaceutical purchase prices was 44.1% ranging from 0.4% to 92.8% between therapeutic areas and areas of indication. There was a significant positive correlation between tender savings and the number of competitors participating in the tender, and a significant negative correlation between tender savings and the number of days since market authorization.

**Conclusions:**

This study finds analogue tenders to be similar in effect and mechanism to competitive tenders in markets for generic medicine and biosimilars. It supports the increasing number of empirical findings that competitive tendering has a high potential to generate substantial savings on healthcare budgets.

## Background

Public expenditure on healthcare absorbs a significant and growing share of many countries' resources. Latest figures from the Organisation for Economic Co-operation and Development (OECD) shows that health spending in OECD countries has grown from around 7.9% in 2003 to 9.7% in 2020 measured as a share of gross domestic product (GDP) [1]. This level and trend in expenditure is influenced by a range of factors affecting both the supply and demand for healthcare including the size and ageing of the population, increasing prevalence of non-communicable diseases, and medical innovation [2, 3].

More than 70% of healthcare spending across OECD countries is funded from public sources [4]. To counteract the increasing financial pressure on public expenditures on health, a vast number of cost-containment policies and instruments have been proposed and recommended by organizations such as the World Health Organization (WHO) [5], the European Union (EU) [6], and The World Bank [7]. A large focus in countries’ cost-containment policies is placed on pharmaceuticals, and most countries today regulate pharmaceutical prices and demand for consumption of pharmaceuticals [8–10].

One of the most effective cost-containment instruments is competitive tenders on pharmaceuticals. Competitive tendering is used when equivalents for a specific medicine are available and can be defined as: “The acquisition of pharmaceuticals based on a competitive bidding process where the contract is granted to the pharmaceutical supplier who offered the best bid following strict criteria” [11]. Competitive tenders are typically conducted to select the most cost-efficient supplier(s) of a particular product and to minimize and fix the purchasing price for the duration of a specific contract period. It is effective in reducing purchase prices because it creates competition among producers participating in the tender, and because it shifts market power towards the buyer [12–15].

While tenders can reduce acquisition costs, they may also expose the healthcare system to risks of negative consequences. Such negative consequences can stem from poor tender practice by the policy makers and purchasers, and from unwanted market responses from the pharmaceutical companies. Poor tender practice includes non-transparent tender practices, a lack of consistency, unclear tender award criteria, a focus on lowest price only, single-winner tendering, and a lack of impact monitoring [11, 16]. Risks of unwanted behavior from pharmaceutical companies includes reduced investments or withdrawal from markets, risk of drug shortages, quality trade-offs, and potentially negative health consequences for patients [17, 18]. Empirical evidence shows considerable reductions in purchase prices on pharmaceuticals [11, 19–22], and limited signs of negative long-term effects on market competition [16, 23, 24].

Most OECD countries use competitive tendering for purchasing pharmaceuticals to some extent, however mostly in hospital settings on mature markets for generic medicine [3, 19, 24]. Generic medicine markets are probably the most relevant area for competitive tenders because the markets typically have one originator with a branded product, and after patent expiration, several generic manufacturers with lower marginal production costs that may push down prices [3, 21].

Recently, competitive tendering has been applied with positive results outside the area of generic medicine. This includes biosimilar drugs, where empirical experiences show tender outcomes and mechanisms comparable to generic medicine tenders [25–27].

In Denmark, a model for competitive tenders on analogue substitutable pharmaceuticals was formally implemented with the establishment of the Danish Medicines Council (DMC) in 2017 [28, 29]. Analogue competition is broadly defined by the DMC as therapeutic areas where several pharmaceuticals with similar effect are available on the market and make competitive exposure possible based on treatment guidelines [30–32]. The purpose of the model is to equate pharmaceuticals within different therapeutic areas based on their clinical effectiveness, thus making them analogue substitutable. Before 2017, analogue competition on hospital pharmaceuticals was practiced for 7 years through the Council for the Use of Expensive Medicine (RADS). The model stands on a close cooperation between the procurement body (Amgros), the health technology assessment body (DMC), and the hospitals owners (i.e., the five Danish regions). A qualitative evaluation of the model was carried out in 2019 by Oxford Research, who finds the model capable of producing large savings on public budgets for pharmaceuticals [30]. Yet no quantitative evaluation of competitive tenders on analogue pharmaceuticals has been carried out in Denmark or elsewhere.

Most international research literature on pharmaceutical tendering is exploratory in nature. Remarkably little empirical research has been conducted to date probably because data are difficult to collect (typically confidential). To our knowledge, the Danish competitive tendering system specially designed for analogue competition on hospital pharmaceuticals is internationally unique [29].

The purpose of this study is therefore to describe and evaluate the experiences from competitive tenders on analogue substitutable pharmaceuticals in Denmark in the period 2017–2020.

## Methods

### Setting

The Danish hospitals are publicly owned and run by the five Danish regions, and all in- and out-patient services are delivered free of charge to citizens. There are no charges for medicine provided to patients at the hospital. Almost all hospital pharmaceuticals (approximately 98%) are purchased by the regions’ central buyer organization, Amgros, who carries out tenders and negotiations with producers and wholesalers. Pharmaceutical pricing is free in Denmark, but historically there have been several attempts to control prices [31]. At the national level, there is a tradition for voluntary price-cap agreements made by the Danish Association of the Pharmaceutical Industry (LIF), the Ministry of Health and the representative organization Danish Regions for both reimbursed medicines and hospital-only medicinal products. The main element in these agreements is that the prices after market introduction should not be increased over time except for defined inflation adjustments. In 2018, the Danish Government also launched the establishment of a statutory external reference price system for prescription and hospital-only pharmaceuticals not covered by these voluntary price-cap agreements. At the regional level, different initiatives to reduce the level of expenditures on pharmaceuticals led to the establishment of the DMC [29]. Since 2017, companies must now apply for a recommendation at the DMC for their pharmaceutical to become possible standard treatment in Danish hospitals. The DMC conducts the health technology assessment on new pharmaceuticals and new indications for existing pharmaceuticals, i.e., they make the decision whether a new pharmaceutical should be recommended as standard treatment in Danish hospitals. This assessment is based on a systematic literature search and an economic evaluation submitted by the manufacturer to document that the price of the pharmaceutical is reasonable in relation to the added value for the patients. The DMC also make new regional treatment guidelines to hospitals for therapeutic areas where pharmaceuticals may be analogue substitutable [31]. The latter is not guided by economic evaluations, but on the judgement by the DMC that alternative pharmaceuticals on the market have ‘similar effect’ for patients. This principle of similar effect is the foundation for Amgros’ analogue national tenders that covers all public hospitals in Denmark. For each analogue tender, the DMC decides in advance the market share for the winner (typically 70–80%). This arrangement is made to preserve alternative treatment options in clinical practice, and to keep all competitors on the Danish market. A tender contract typically applies for a 1-year time horizon, but with an option for Amgros to prolong the contract with 1 year. The treatment guidelines are updated to include the results from the analogue tender, and the contract is enforced through collaboration between Amgros, the hospital pharmacies and the regional drug committees [28, 30].

### Design and data

We obtained data from Amgros on all analogue competitive tenders carried out by the DMC from its foundation on January 1, 2017, to October 9, 2020 [32]. These analogue tenders constituted around 43% of Amgros’ annual purchase of hospital medicine. The areas put out for analogue tender in this period were chosen by the DMC. No information was provided about the selection criteria used by the DMC.

Data were provided in excel format on a USB stick after signing the research agreement. The material included the tender results from nineteen tenders covering eight different therapeutic areas at Anatomical Therapeutic Chemical classification system (ATC) level five (some therapeutic areas had multiple indication areas put out for analogue tender). The dataset contained information on the specific pharmaceuticals that were included in the tenders, application areas in the therapeutic area, tender price, number of competitors bidding, and promised market share for the winner. Tender prices were confidential. Amgros also provided data on the total annual consumption of each of the nineteen winning pharmaceuticals from the period May 2020 to April 2021.

Date of marketing authorization for each winning pharmaceutical was obtained from European Medicines Agency (EMA) [33]. Official list prices were obtained from Danish Health Data Authority (DHDA) for the period from marketing authorization until the date of the tender [34].

### Analyses

We applied standard economic theory on tendering as analytical framework [12–15]. The discount rate of the winning pharmaceutical for each of the nineteen tenders was calculated by comparing confidential prices with official list prices per defined daily dose (DDD) or standard treatment. For this part, we were able to include and verify the comparisons performed by Amgros. An estimate of the annual gross savings in Danish currency (DKK) was made as a percentage of consumption values for each pharmaceutical from May 2020 to April 2021 (i.e., assuming that the tender discount for each pharmaceutical had been deducted from official list prices). We calculated the annual price decrease for each pharmaceutical from the EMA authorization date until date of tender to separate out general price movements of each pharmaceutical. The number of days between EMA authorization and tender was taken as a measure of product maturity. The number of competitors was used as a measure of competition intensity. Finally, the promised market share and pharmaceutical spending were used as indicators of a bidder’s strategic consideration regarding the expected value of winning the bid.

### Statistics

We calculated univariate descriptive statistics for all the variables including mean, median, minimum, and maximum as well as standard deviation. To assess associations, we calculated pairwise correlations between all our variables. Finally, to elaborate on the pairwise associations we made a multiple regression analysis (ordinary least squares) of the discount rate on all the remaining variables [35].

## Results

Average annual saving on hospital pharmaceutical purchase prices was 44.1% obtained through competitive analogue tender (Table 1). The savings ranged from 0.4% to 92.8% between therapeutic areas and areas of indication. Measured in DKK, the total savings on hospitals pharmaceuticals were estimated to approximately DKK 1.114.037.700 per year.

**Table 1:** Descriptive statistics of the sample

Variable	*n*	Mean	Median	Minimum	Maximum	Standard deviation
Percentage discount rate	19	44.10	44.31	0.40	92.84	30.93
Annual percentage price decrease	19	8.16	2.42	0	72.58	16.91
Number of days since authorization	19	1666.37	894	313	7801	1878.54
Number of competitors	19	4.89	3	1	14	4.62
Promised market share for the winner	19	84.34	80	60	100	10.13
Pharmaceutical spending (million DKK/year)	19	44.01	15.91	0.23	161.27	50.68

**Fig. 1:**
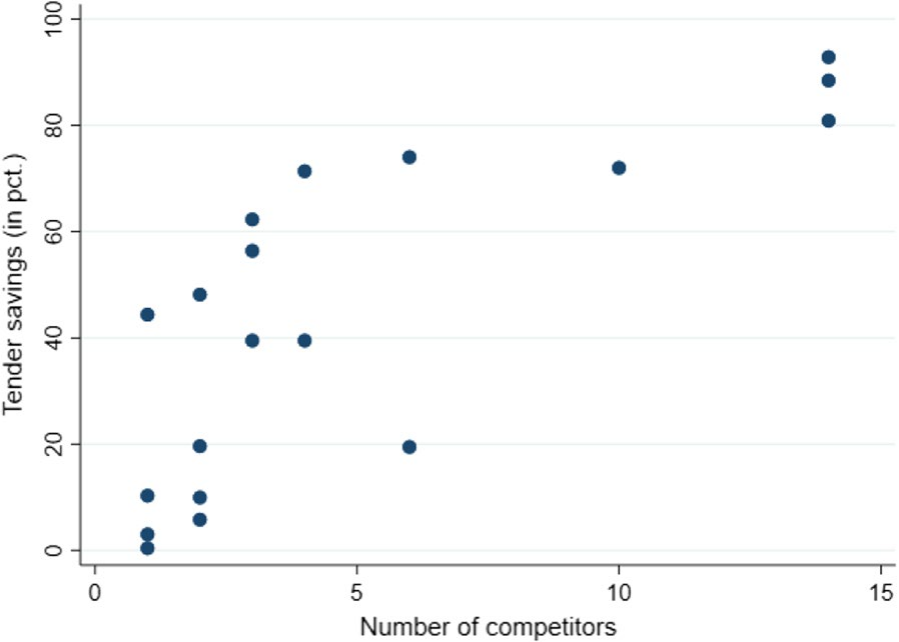
Tender savings compared to the number of competitors. Tender savings are calculated as the official list price minus the confidential discount obtained on the winning product through the tender. The number of competitors is the number of bidders in the tender

**Fig. 2:**
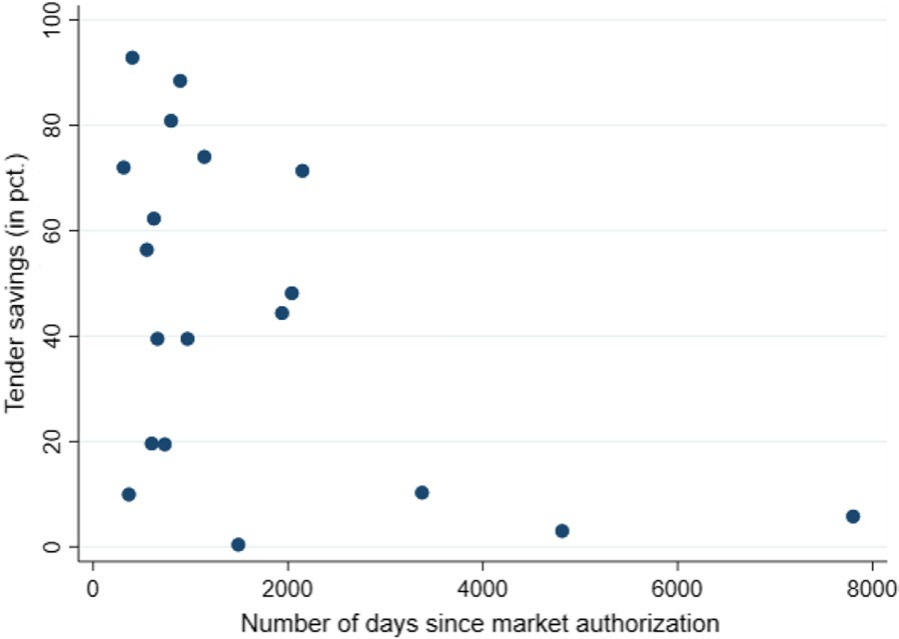
Tender savings compared to the number of days since market authorization. Tender savings are calculated as the official list price of the winning product price minus the confidential discount obtained through the tender. The number of days since market authorization is the number of days the winning product has been on the Danish market

There was a positive and statistically significant correlation between tender savings (in %) and the number of competitors participating in the tender (Fig. 1). There was a negative and statistically significant correlation between tender savings and the number of days since market authorization (EMA approval) (Fig. 2). No other statistically significant correlations were found (Table 2). The average annual decline in public list prices before tenders was 8.2% (4.5% if one pharmaceutical is removed).

**Table 2:** Pairwise correlation and (*p*-values)

	Percentage discount rate	Annual percentage price decrease	Number of days since authorization	Number of competitors	Promised market share for the winner	Pharmaceutical spending (million DKK/year)
Percentage discount rate	1					
Annual percentage price decrease	0.26 (0.28)	1				
Number of days since authorization	− 0.49 (0.03)	− 0.27 (0.26)	1			
Number of competitors	0.77 (< 0.00)	0.33 (0.16)	− 0.39 (0.10)	1		
Promised market share for the winner	− 0.25 (0.30)	0.06 (0.80)	0.11 (0.65)	− 0.22 (0.37)	1	
Pharmaceutical spending (million DKK/year)	− 0.31 (0.18)	− 0.02 (0.94)	0.16 (0.52)	− 0.37 (0.12)	0.08 (0.75)	1

The regression analysis explained approximately 72% of the variation in the savings at tenders (Table 3). Controlling for promised market share, annual turnover (total consumption of the pharmaceutical in Denmark the year after the tender), number of days since EMA approval, annual decline in public list price before the tender, the number of competitors was still positively and significantly related to tender savings (in %). Thus, each additional competitor in the tender provides an expected extra saving of approximately 5%.

**Table 3:** Regression results from analyzing percentage discount rate

	Coefficient	Robust standard error	*t*
Constant	49.87	67.30	0.81
Annual percentage price decrease	− 0.04	0.18	− 0.21
Number of days since authorization	− 0.004	0.002	− 2.08
Number of competitors	4.46	1.04	4.29
Promised market share for the winner	− 0.24	0.67	− 0.36
Pharmaceutical spending (mio. DKK)	− 0.02	0.11	− 0.18
*n*	19		
*R*^2^	0.72		

## Discussion

This study is the first to quantitatively assess the effect of competitive analogue tenders on pharmaceuticals for public hospitals. Our results indicate that analogue tenders are remarkably similar in effect and mechanism to competitive tenders in hospital markets for generic medicine and biosimilars.

The calculated cost savings in Danish hospitals were 44% (0.4–92.8%) on pharmaceuticals purchased through competitive analogue tenders. In monetary terms, the annual savings were estimated to approximately DKK 1.1 billion corresponding to 6–7% of the annual total hospital consumption of pharmaceuticals in Denmark measured in pharmacy purchase prices (PPP) [36]. These figures should, however, be interpreted with caution and only as ‘gross’ savings, because Amgros would alternatively have obtained some rebates through other means of negotiation. Nevertheless, the estimated savings from the tenders were undoubtedly larger than what could otherwise have been obtained either through market development over time [including the voluntary price-cap agreement between the Government and the Danish Pharmaceutical Industry (LIF)], or the discounts Amgros normally are able to obtain on patent protected pharmaceuticals through negotiation [36]. The estimated savings only include the discounts on the winning pharmaceutical (i.e., approximately 80% of consumption corresponding to the promised market share for winning a tender). Differences in administration cost and cost-effectiveness between pharmaceuticals were not included in the calculation.

The results should not be interpreted as a causal analysis of competitive analogue tenders. The therapeutic areas in the sample were selected by the DMC, and selection criteria are unknown. We cannot conclude that analogue tenders create possibilities for large public savings in general, but it does in a number of situations.

Results also show a huge variation in savings ranging from 0.4% to 92.8%. This appears to be a larger variation compared to tenders on generics and biosimilars [23]. One possible explanation for a larger variation in savings on analogue tenders is strategic issues in the market, i.e., a question of product differentiation [37]. This indicates differences between analogue and generic medicine markets. According to standard microeconomic theory, generic manufacturers (copy producers) in competitive environments can be assumed to have similar marginal costs of production and cannot eliminate each other through market competition [10, 13, 14]. Analogue competitors per definition have different products (but with similar effect). The manufacturers of analogue pharmaceuticals use different production technologies, and their production cost may accordingly be very different. Competitive analogue tendering could potentially have other consequences for long-term market competition compared to tendering in markets for generics. This study therefore suggests that the effect of competitive analogue tenders may exhibit larger variation in results compared to generic markets and biosimilars. However, this should be further investigated.

This study supports the increasing number of findings that competitive tendering has a high potential to generate substantial savings in OECD health systems [5–7]. Public tendering, however, puts high demands for good governance upon the organizations responsible for carrying out the processes [18, 38] and for that reason, tendering for pharmaceuticals is not used to its full potential [3]. Tendering needs antitrust scrutiny to secure competition and compliance with rules and principles [39]. An inefficient bureaucracy can represent a major obstacle to efficient tendering due to problems with lack of incentives among bureaucrats and risks of opportunism [10, 40]. OECD estimates that up to 25% of public procurement (on pharmaceuticals, devices, equipment, etc.) is lost to corrupt practices, fraud, and poor public procurement practices [6]. The number of observations in this sample was also limited and shows that even with a clear public strategy to obtain savings through analogue tenders, it is difficult to reach a high number of tenders because it requires a great bureaucratic effort [28–30].

There is no suggestion in economic theory that competitive tendering will lead to lower spending levels in the public healthcare system as a whole [15]. The savings obtained from analogue tenders will probably not be converted into tax reductions for the Danish citizens, but more likely be used to make room for other purchases and other healthcare activities. Even though, the total budget for the DMC is only around DKK 60 million annually. The estimated annual savings from analogue tenders exceeds this amount more than fifty times. This clearly illustrates the economic potential from tenders, let alone the importance of efficient public governance.

## Conclusion

This study shows that competitive tendering on analogue substitutable pharmaceuticals can provide large savings on public healthcare budgets. Annual savings on hospital pharmaceuticals purchase prices were 44.1% obtained through competitive analogue tenders. For each additional competitor participating in a tender an extra saving of approximately 5% was obtained. Potential negative long-term effects of the analogue competition program, e.g., companies choosing not to participate in the tender or withdrawing their products from the market should be further investigated in the future.

## Data Availability

The data that support the findings of this study may be available at request from Amgros I/S, https://amgros.dk/kontakt/, but restrictions apply to the availability of these data, and so they are not publicly available.

## Abbreviations

OECD: Organisation for Economic Co-operation and Development
WHO: World Health Organization
EU: European Union
DMC: Danish Medicines Council
EMA: European Medicines Agency
DKK: Danish currency
